# Can severity of a humanitarian crisis be quantified? Assessment of the INFORM severity index

**DOI:** 10.1186/s12992-023-00907-y

**Published:** 2023-01-31

**Authors:** Velma K. Lopez, Angeliki Nika, Curtis Blanton, Leisel Talley, Richard Garfield

**Affiliations:** 1 U.S. Centers for Disease Control and Prevention, Atlanta, GA USA; 2 ACAPS, Geneva, Switzerland

**Keywords:** Humanitarian crisis, Severity, Factor analysis

## Abstract

**Background:**

Those responding to humanitarian crises have an ethical imperative to respond most where the need is greatest. Metrics are used to estimate the severity of a given crisis. The INFORM Severity Index, one such metric, has become widely used to guide policy makers in humanitarian response decision making. The index, however, has not undergone critical statistical review. If imprecise or incorrect, the quality of decision making for humanitarian response will be affected. This analysis asks, how precise and how well does this index reflect the severity of conditions for people affected by disaster or war?

**Results:**

The INFORM Severity Index is calculated from 35 publicly available indicators, which conceptually reflect the severity of each crisis. We used 172 unique global crises from the INFORM Severity Index database that occurred January 1 to November 30, 2019 or were ongoing by this date. We applied exploratory factor analysis (EFA) to determine common factors within the dataset. We then applied a second-order confirmatory factor analysis (CFA) to predict crisis severity as a latent construct. Model fit was assessed via chi-square goodness-of-fit statistic, Comparative Fit Index (CFI), Tucker-Lewis Index (TLI), and Root Mean Square Error of Approximation (RMSEA). The EFA models suggested a 3- or 4- factor solution, with 46 and 53% variance explained in each model, respectively. The final CFA was parsimonious, containing three factors comprised of 11 indicators, with reasonable model fit (Chi-squared = 107, with 40 degrees of freedom, CFI = 0.94, TLI = 0.92, RMSEA = 0.10). In the second-order CFA, the magnitude of standardized factor-loading on the ‘societal governance’ latent construct had the strongest association with the latent construct of ‘crisis severity’ (0.73), followed by the ‘humanitarian access/safety’ construct (0.56).

**Conclusions:**

A metric of crisis-severity is a critical step towards improving humanitarian response, but only when it reflects real life conditions. Our work is a first step in refining an existing framework to better quantify crisis severity.

**Supplementary Information:**

The online version contains supplementary material available at 10.1186/s12992-023-00907-y.

## Background

Humanitarian crises present a multitude of possible harmful health consequences for individuals [1]. When populations are displaced, new settlements can have poor housing and sanitation conditions, leading to increased prevalence of acute infectious diseases, such as respiratory and enteric illnesses [2, 3]. Disruption of food systems can result in acute malnutrition [4, 5] as well as further chronic malnutrition [6]. In addition, interruption of health services limits the management of chronic diseases [7], access to sexual and reproductive health care [8], and distribution of immunizations among children [9]. Mental health disorders, namely post-traumatic stress disorder, depression, and anxiety, are common among the displaced [10]. While each of these morbidities are harmful on their own, they often interact, worsening overall wellbeing [1], and playing a role in the larger ecosystem that affects high mortality rates among crisis-affected people [11]. Moreover, the impacts of humanitarian crises go beyond individual well-being to negatively influence communities, society, and the environment [12]. Given the potentially devastating and longstanding impact of a humanitarian crisis, it is critical to provide aid where it is needed most.

The need for humanitarian assistance is great; yet there is limited funding available for response. For example, in 2020, the United Nations Office for the Coordination of Humanitarian Affairs (UNOCHA) estimated that there were 166.5 million people requiring humanitarian assistance and a gap of $14 billion (USD) in aid funding [13]. With limited funding, it is imperative to assess which populations are in the greatest need and allocate resources accordingly. Currently, the United Nations (UN) system uses the crisis metric *number of people in need (PIN) of humanitarian assistance* to guide aid allocation. Because crises are diverse, the definition of PIN is non-specific [14] and the estimation is based on non-standardized data collection [15]. Thus, using PIN as a basis of allocating resources may be limited. One step towards improving aid allocation is shifting the paradigm away from asking “how many people are in need?” and towards, “how bad are their needs?”. Accordingly, a systematic metric of crisis severity, that is “how bad is it?”, would reflect needs in ongoing crises and predict severity if conditions change [16]. Applying a component measurement of crisis severity to aid allocation should help align resource distribution with core humanitarian principles: humanity, impartiality, independence, and neutrality.

Developing a metric to quantify the severity of a crisis is challenging. First and foremost, humanitarian crises are diverse and evolving events. Of the metrics that can be applied to a wide array of crises, most are designed for intra-country assessment of severity (e.g., the UNOCHA’s Humanitarian Needs Comparison Tool [17] or Kandeh et al.’s assessment of crisis-related vulnerability in Yemen [18]). While it is useful to assess geographic disparities, the need for humanitarian assistance is often based on estimates of overall crisis severity. For example, Bayram et al.’s 2012 Public Health Impact Severity Scale recommends using expert opinion to rank 12 indicators from the Sphere Project “Minimum Standards”, with the final severity score reflecting a weighted sum of the ranks [19]. This framework, however, has yet to be implemented as the authors state limited availability of timely data. Eriksson et al. proposed a similar approach of ranking and summing key variables, but conceptualized severity as more holistic predictor of humanitarian need by drawing on psychological theory and ranked variable importance based on presence of the indicator in the literature [20]. Like the Public Health Impact Severity Scale, use of their model has not been widespread.

The current model used to quantify crises severity is the INFORM Severity Index, a metric based on comparable data drawn from publicly available sources [21]. Developed via partnerships and through consensus building among experts, the index uses a conceptual framework that describes crisis severity as a complex, multi-factorial construct after the immediate, emergency phase of crisis. The index is used by policymakers to set or justify priorities for providing humanitarian support, bring attention to unknown crises, and to monitor crisis trends. However, the model has not to date undergone statistical review.

We seek to critically evaluate the overall index model structure and assess the relationships between indicators. The INFORM Severity Index database, which was publicly available under the name ‘Global Crisis Severity Index (GCSI)’, included 172 unique crises in the version released in December 2019. These crises were either ongoing as of November 30, 2019, or had occurred earlier in 2019. The INFORM model inputs 35 unique indicators across three pillars (‘impact of the crisis’, ‘complexity of the crisis’, ‘conditions of the people’) to estimate crisis severity (see Table 1 for data and definitions and Additional file 1: Appendix 1 for details regarding the GCSI construction). Because this is the most commonly used data source for humanitarian stakeholders to assess crisis severity, our objective is to determine if the entire model or a subset of its components could be used to estimate crisis severity through a score. Importantly, the GCSI dataset tracks diverse crises and analysis of it provides insight into severity of a wide range of emergency events. Here, we applied factor analysis, a method commonly used for data reduction of highly correlated and grouped data. This review is an attempt to generate a more robust estimate of crisis severity.

**Table 1:** Global Crisis Severity Index (GCSI) pillar, which we consider latent constructs, presented alongside the indicators within each construct, definition, whether the indicator is crisis related, and if not, when the data are routinely collected. All indicators, and their references, are further described within the GCSI spreadsheet under the sheet titled “Indicator Metadata”: https://data.humdata.org/dataset/inform-global-crisis-severity-index

GCSI Latent Constructs	Indicators	Definition	Type of Indicator	Frequency of Collection
Impact of the Crisis	Landmass affected - absolute	Total number of square kilometers affected by the crisis	Crisis	N/A
	Landmass affected - relative	Percent of square kilometers affected by the crisis	Crisis	N/A
	People living in the affected area - absolute	Total number of people living in the affected area	Crisis	N/A
	People living in the affected area - relative	Percent of people living in the affected area	Crisis	N/A
	People affected - absolute	Total number of people affected by the crisis	Crisis	N/A
	People affected - relative	Percent of people affected by the crisis	Crisis	N/A
	People displaced - absolute	Total number of crisis related displaced people	Crisis	N/A
	People displaced - relative	Percent of crisis related displaced people	Crisis	N/A
	^a^Number of people ill	Total number of crisis related ill people	Crisis	N/A
	^b^Number of people injured	Total number of crisis related injured people	Crisis	N/A
	^c^Number of fatalities	Total number of crisis related fatalities	Crisis	N/A
Complexity of the Crisis	Corruption perception index	The CPI scores and ranks countries/territories based on how corrupt a country’s public sector is perceived to be. It is a composite index, a combination of surveys and assessments of corruption, collected by a variety of reputable institutions.	Non-crisis	Yearly
	Rule of law (WGI)	Rule of law, a variable included in the Worldwide Government Indicators (WGI) captures perceptions of the extent to which agents have confidence in and abide by the rules of society, and in particular the quality of contract enforcement, property rights, the police, and the courts, as well as the likelihood of crime and violence.	Non-crisis	Yearly
	Rule of law (BTI)	The Bertelsmann Stiftung’s Transformation Index (BTI) analyzes and evaluates the quality of democracy, a market economy and political management in 129 developing and transition countries. It measures successes and setbacks on the path toward a democracy based on the rule of law and a socially responsible market economy. It also entails an evaluation of the rule of law including the separation of powers and the prosecution of office abuse.	Non-crisis	Every 2 years
	Freedom in the World	Freedom in the World is Freedom House’s flagship annual report, assessing the condition of political rights and civil liberties around the world. It is composed of numerical ratings and supporting descriptive texts for 195 countries.	Non-crisis	Once (in 2017)
	Total killed in all crisis	Number killed in the crisis affected area in the last three months	Crisis	N/A
	Conflict intensity	The HIIK’s annual publication Conflict Barometer describes the recent trends in global conflict developments, escalations, de-escalations, and settlements.	Non-crisis	Yearly
	Gender inequality	The Gender Inequality Index (GII) reflects gender-based disadvantages in three dimensions—reproductive health, empowerment and the labour market. The value of GII range between 0 to 1, with 0 being 0% inequality, indicating women fare equally in comparison to men and 1 being 100% inequality, indicating women fare poorly in comparison to men.	Non-crisis	Yearly
	Income gini coefficient	Gini index measures the extent to which the distribution of income or consumption expenditure among individuals or households within an economy deviates from a perfectly equal distribution. A Gini index of 0 represents perfect equality, while an index of 100 implies perfect inequality.	Non-crisis	Yearly
	Ethnic fractionalisation	Ethnic fractionalisation Index is calculated using a simple Herfindahl concentration index from Ethnic Power Relations (EPR) Dataset.	Non-crisis	Yearly
	Size of excluded ethnic groups	The Minorities at Risk (MAR) project monitors and analyzes the status and conflicts of politically-active communal groups in all countries. The focus of the MAR project has been “minorities at risk.	Non-crisis	Once (from 2004 to 2006)
	Empowerment	This is an additive index constructed from the Foreign Movement, Domestic Movement, Freedom of Speech, Freedom of Assembly & Association, Workers’ Rights, Electoral Self-Determination, and Freedom of Religion indicators. It ranges from 0 (no government respect for these seven rights) to 14 (full government respect for these seven rights).	Non-crisis	Yearly (1981-2011)
	BTI - Democracy status	The Bertelsmann Stiftung’s Transformation Index (BTI) analyzes and evaluates the quality of democracy, a market economy and political management in 129 developing and transition countries. It measures successes and setbacks on the path toward a democracy based on the rule of law and a socially responsible market economy. It also entails an evaluation of the rule of law including the separation of powers and the prosecution of office abuse.	Non-crisis	Every 2 years
	Crisis affected groups	Number of different types of affected population groups, based on categories of the IASC Humanitarian profile COD 2012. The final value represents a count of types of affected group, at the lowest level of the humanitarian profile page 5 of the document.	Crisis	N/A
	Impediments to entry into country (bureaucratic and administrative)	This indicator refers to the general access of international actors into the country. It refers to registration, accreditation and visa policies, provision of taxes or fees on activities or goods; policies related to import and logistics; visa or accreditation delays or denial; discretional registration or visas by authorities, and presence of humanitarian organisations and workers in the country being allowed to operate.	Crisis	N/A
	Restriction of movement (impediments to freedom of movement and/or administrative restrictions)	This indicator refers to the in-country mobility of humanitarian workers in order to reach the affected population and transport relief items. It includes presence of taxes and fines on passage of goods and people, quotas and limits on relief items in specific areas, assistance seized, agencies on hold despite being ready to intervene, checkpoints, or closure of border crossings.	Crisis	N/A
	Interference into implementation of humanitarian activities	This indicator refers to factors such as conditions imposed on the type of aid, or the modality of aid delivery. It includes operational restrictions imposed by government as well as confiscation or diversion of aid.	Crisis	N/A
	Violence against personnel, facilities and assets	This indicator takes into account security incidents involving humanitarian organisations. Incidents include attacks, abduction, execution, kidnapping of workers, and looting of humanitarian warehouses or humanitarian assets.	Crisis	N/A
	Denial of existence of humanitarian needs or entitlements to assistance	This indicator takes into account statements that demonstrate a recognition or denial of needs of a population or the rights of minorities, and any discrepancy between the reported humanitarian needs and official statements.	Crisis	N/A
	Restriction and obstruction of access to services and assistance	This indicator refers to the affected population’s perspective. It assesses whether people are prevented from reaching aid or services – through various restrictions, such as prevention of the crossing of borders to seek refuge, administrative barriers, or requirements to have specific documents. Sieges, roadblocks, curfews, and harassment are be considered.	Crisis	N/A
	Ongoing insecurity/hostilities affecting humanitarian assistance	This indicator takes into account the presence of ongoing hostilities or violence that affects humanitarian operations, leading to decisions to divert or suspend aid, or to evacuate or modify operations.	Crisis	N/A
	Presence of mines and improvised explosive devices	This indicator looks into how the presence of landmines or Unexploded Ordnance (UXOs) might hinder humanitarian access.	Crisis	N/A
	Physical constraints in the environment (obstacles related to terrain, climate, lack of infrastructure, etc.)	This indicator looks into seasonal events or weather conditions as well as preexisting infrastructure. Status of roads, bridges, and airfields are also considered, along with communications and logistical constraints such as lack of fuel or assets hampering physical accessibility to people in need.	Crisis	N/A
Conditions of the People^d^	Total People in Need	The total number of people in need in each crisis. The total number of people in need equals the sum of people experiencing moderate, severe, and extreme humanitarian conditions.	Crisis	N/A
	Current humanitarian conditions of total population in the affected area	The conditions and status of the people affected, including information about the distribution of severity (i.e. the number of people in each category of severity within a crisis). The humanitarian conditions severity is distributed in 5 levels, each of them is defined separately.	Crisis	N/A

^a^82% observations were missing

^b^71% observations were missing

^c^27% observations were missing

^d^Indicators are estimated by first classifying the population into five levels of humanitarian conditions:

Level 1: None/Minor humanitarian conditions: People are facing none or minor shortages or/and accessibility problems regarding basic services. People are able to meet basic needs, such as food, health, shelter, and wash, without having to apply irreversible coping strategies. There may be some needs but are not life-threatening

Level 2: Stressed humanitarian conditions: People are facing some shortages or/and some availability and accessibility problems regarding basic services. Needs are higher but are still not life-threatening. The affected population can meet their needs by applying copying strategies. There may exist localised/targeted incidents of violence and/or human rights violations

Level 3: Moderate humanitarian conditions: People are facing shortages and/or availability and accessibility problems regarding basic services which is causing discomfort and/or high level of suffering, but conditions are not life-threatening. Significant service gaps are visible, and people are marginally able to meet needs only with irreversible coping strategies. People may also face malnutrition. There may be physical and mental harm to populations. The need for humanitarian assistance is more likely

Level 4: Severe humanitarian conditions: People are facing life-threatening conditions and significant shortages and/or availability and accessibility problems causing high level of suffering and irreversible damages. People face severe food consumption gaps and have started to deplete their assets or already face an extreme loss of assets. This may result in very high levels of acute malnutrition. Presence of irreversible harm as well as widespread grave violations of human rights and excess mortality. Humanitarian assistance is required

Level 5: Extreme humanitarian conditions: People are facing extreme shortages or availability and accessibility problems regarding basic services. There is widespread mortality as a direct result of current condition. People may face a complete lack of food and starvation is likely. Basic needs are not being met and destitution is evident. Acute malnutrition may be widely reported. Presence of irreversible harm as well as widespread grave violations of human rights and excess mortality. Humanitarian assistance is required

*N/A* not applicable

## Methods

### Data

We analyzed data from the beta version of the INFORM Severity Index database, which was publicly available under the name ‘Global Crisis Severity Index (GCSI)’ and released in December 2019 [22]. We extracted data from 172 unique global crises that were either ongoing as of November 30, 2019, or had occurred earlier in 2019. Additional file 1: Appendix 1 describes how the INFORM Severity Index is calculated.

### Measures

The GCSI uses a total of 35 ordinal indicators to represent three pillars (impact of the crisis, complexity of the crisis, conditions of the people), which we consider latent constructs (Table 1). Each ordinal indicator is scored based on continuous variables. The first construct, ‘the impact of the crisis’, is comprised of 11 indicators, all of which are ordinal versions of data collected from the specific crisis. The second construct, ‘the complexity of a crisis’, is comprised of 22 indicators. Of these indicators, 12 are publicly available indices; one is an ordinal version of data collected from the specific crisis; and the remaining nine indicators reflect qualitative information that is given a quantitative score. The final construct, ‘conditions of the people’, has two indicators, each of which uses estimates of the number of people in need of humanitarian assistance for the given crisis. Spearman’s rank correlation coefficients for all indicators are presented in Additional file 1: Appendix 1. From the 35 GCSI indicators, we removed three indicators that had more than 25% of observations missing.

### Analytical approaches

Modeling a construct such as ‘severity’ requires leveraging information from multiple indicators. Any approach that does not account for correlation between the indicators will likely result in imprecise final estimates. Thus, our analytical framework uses structural equation modeling to predict crisis severity. This method explicitly includes measurement error for each indicator, assessment of model fit – both overall and at the indicator level -, and prediction from optimal combinations of indicators. The overall goal of the analysis is to deduce causal relationships by accounting for correlation coefficients. To do so, we have a two-step approach. First, we used exploratory factor analysis (EFA) to identify patterns of grouping among the indicators. This step provided insight on whether the data supported the GCSI pillar construction. We then applied confirmatory factor analysis (CFA) to test whether the identified relationships from the EFA were statistically meaningful and if the multiple latent constructs were inter-related (as hypothesized by the GCSI pillar construction).

#### Exploratory factor analysis

We evaluated the relationships between 32 indicators in the GCSI conceptual framework through EFA. Based on an initial scree plot, we employed four maximum likelihood EFA models, ranging from 3- to 6 -factor solutions, each with an oblimin rotation, that is, correlation was permitted between factors [23]. Missing values were imputed with the indicator median within all EFAs. We evaluated the models for the following characteristics: sums of squared loadings greater than 1.0 for each factor; factors that contribute to at least 10% to the overall variance; and collective contribution of at least 60% of the overall variance. Next, we reviewed the indicator factor loadings to identify latent constructs within the dataset.

Using the information learned from the EFA models, we removed indicators from the dataset if they did not provide unique information to identified factors as their inclusion in a final score could lead to either bias or imprecision. Indicators were removed if they had factor loadings less than 0.30 or cross-loaded onto more than one factor with a loading less than 0.20, or if cross-loadings had values in opposite directions (for example, 0.37 and − 0.33).

#### Confirmatory factor analysis

With the reduced dataset and using standardized indicators, we applied a full information maximum likelihood CFA to model crisis severity. First, we built first-order CFAs with relationships identified in the EFA. We removed indicators from the CFAs if they had residuals greater than 0.10 with indicators on different latent constructs. We also added covariances between indicators on the same latent construct if their residual correlation was greater than 0.10. Finally, we added a second-order latent construct to the model, which represented ‘crisis severity’. Model fit was assessed via chi-square goodness-of-fit statistic, Comparative Fit Index (CFI), Tucker-Lewis Index (TLI), and Root Mean Square Error of Approximation (RMSEA). Acceptable model fit was evaluated using recommended cut-offs characterized as CFI and TLI greater than 0.90 and RMSEA less than 0.08 [24].

We also estimated values for the latent crisis severity variable (i.e., factor scores) based on the factor loadings in the second-order CFA. Latent severity scores were normalized to range from zero to one.

We conducted several subanalyses to determine the robustness of the overall results. These analyses focused on: incorporating the ‘people in need of humanitarian assistance’ indicator within the final models (see Additional file 1: Appendix 5); data quality implications (see Additional file 1: Appendix 6); comparison of the modeled scores to the original scores (see Additional file 1: Appendix 7); the role of governance when estimating crisis severity (Additional file 1: Appendix 8); the implication of missing data (see Additional file 1: Appendix 9); and the overall model fit bootstrapped subsamples of the dataset (see Additional file 1: Appendix 10).

Analyses were conducted using the R (version 3.6.2) packages *psych*, *GPArotation*, and *lavvan*; see Additional file 1: Appendix 4 for primary analyses’ R code.

The Human Research Protection Office within the Center for Global Health at the Centers for Disease Control and Prevention reviewed the study and determined it to be non-research.

## Results

### Original GCSI score

**Fig. 1:**
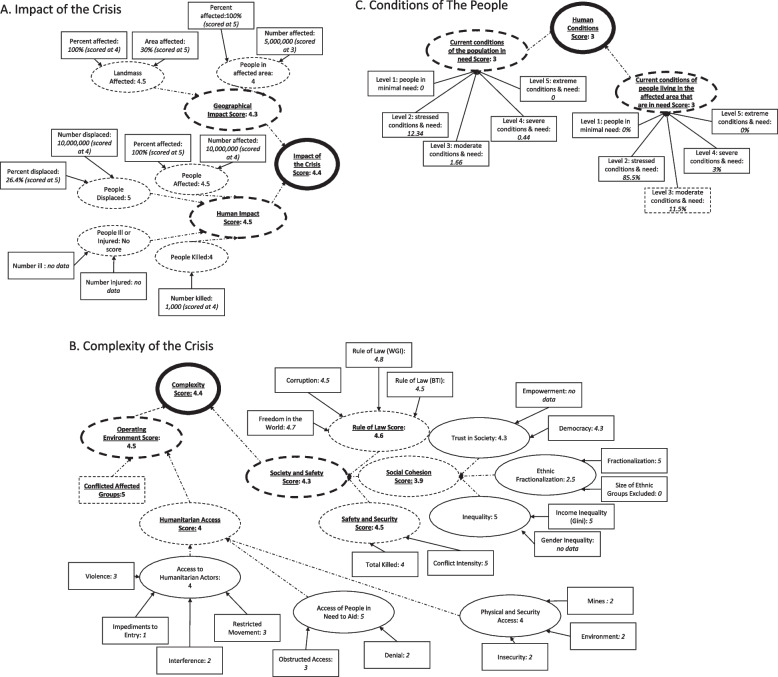
A schematic of the GCSI conceptual framework for the complex crisis in Somalia (coded as *SOM001* in the GCSI database). Each box represents a data point, each oval represents the aggregation of the boxes (or other ovals) preceding it (represented by an arrow), and each circle represents the aggregation into the GCSI pillars. Shapes with dashed values represented aggregated scores of sib-indicators and bold shapes are the aggregated final scores. Panel **A** shows the Impact of the Crisis. Panel **B** shows the Complexity of the Crisis. Panel **C** shows the Conditions of the People. In panel **C**, the Condition of the population in Need Score shows values that are scaled to 1,000,0000 people

The GCSI includes a large number of indicators (Table 1), which reflect both crisis related data and non-crisis related data. The original GCSI score is generated from combining these indicators into pillars, which are then aggregated into a score. For example, the complex emergency in Somalia, coded as *SOM001* in the GCSI database, was classified as having “High Severity” with a score of 4.0 as of November 2019. Qualitatively, there is concurrence between the GCSI score given to the Somali crisis and the nation’s social structure and events that have occurred there; approximately 4 million people needed humanitarian assistance in Somalia in 2019, and millions had been displaced by recurring conflict, insecurity, forced evictions, drought, and floods (see https://www.unocha.org/somalia). The GCSI Severity Score for Somalia was estimated via a weighted mean of the values derived for each pillar: 4.4 (with a 0.2 weight applied) for the Impact of the Crisis, 4.4 (with a 0.3 weight applied) for the Complexity of the Crisis, and 3.0 (with a 0.5 weight applied) for the Conditions of the People. The data feeding into these estimates, and their values, are presented in Fig. 1. Within the figure, each box is a data point, each oval represents the aggregation of the boxes (or other ovals) preceding it (represented by an arrow), and each circle represents the aggregation into the GCSI pillars. Importantly, each pillar calculation, and the calculation of the sub-indicators used in the pillar score, is unique (see Additional file 1: Appendix 1 for details). Briefly, the pillar scores are derived using the following approaches:

- The Impact of the Crisis (Fig. 1A) is the weighted sum of two composite indicators - The Human Impact (weighted at 0.7) and the Geographical Impact (weighted at 0.3). The Human Impact Score is an aggregate of 4 sub-indicators, however, for Somalia, data are only available for 3 components. The Geographical Impact is a mean score generated from two sub-indicators.
- The Complexity of the Crisis (Fig. 1B) is estimated by calculating the geometric mean of two composite indicators – Society and Safety and Operating Environment. The Society and Safety Score reflect a mean of three sub-indicators, each with a different number of data inputs. Missing inputs, such as ‘Gender Inequity’ in Somalia, are ignored during aggregation. The Operating Environment Score is estimated from the average value of a sub-indictor and a data input variable. However, here, the sub-indicator aggregation mostly reflects summation, and is scaled if there are more than two variables that contribute to the sub-indicator.
- Conditions of the People as a Result of the Crisis (Fig. 1C) is the average of two sub-indicators – Current Humanitarian Conditions of the Total Population, and Current Humanitarian Conditions of the Population Affected. Here, the population is ranked into one of five levels: 1. those facing minimal humanitarian need, 2. those in stressed humanitarian conditions and needs, 3. those in moderate humanitarian conditions and needs; 4. those in severe humanitarian conditions and needs, and 5. those in extreme humanitarian conditions and needs. The Current Humanitarian Conditions of the Total Population sub-indicator reflects the sum of people in levels 3-5, which is then ranked. The Current Humanitarian Conditions of the Population Affected sub-indictor, however, is calculated slightly differently. Here, the highest level is taken if the percent of the population affected at that level is greater than 5%. For example, the Somalia crisis is given a value of 3 for this indicator because 11.5% of the population affected fall into level 3 of need.

Overall, Fig. 1 paints an intricate, and convoluted, picture of data relationships used to classify the severity of the Somali crisis. Understanding these relationships sheds insight into how these data can be used to generate severity values.

### GCSI analysis

To guide our analysis, we first examined the following characteristics of the GCSI data frame: correlation between indicators, distributions of indicators, and the proportion of non-missing data. The indicators are highly correlated (see Additional file 1: Appendix 1) within the conceptual GCSI pillars and between them, with approximately 31% of the indicators having correlation coefficients greater than +/− 0.6. The correlation values suggest complex underlying relationships, and inference thereof required a method that accounts for statistical dependencies. Mean and median values of the ordinal scores did not differ greatly for most indicators, suggesting only slightly skewed distributions (Table 2) and the appropriate application of parametric methods. Three indicators had a substantial proportion of missing data (‘Number of people ill’; ‘Number of people injured’; ‘Number of fatalities’), so we removed them from our analysis. Seventy-five to 100% of observations were available for the remaining 32 indicators. The indicators related to people displaced from a crisis had the two lowest number of observations (75 and 77% of total observations), as did the indicator for people in need (80% of total observations).

**Table 2:** Descriptive statistics for Global Crisis Severity Index (GCSI) indicators: Mean, standard deviation, median, range, and total number of observations (n) are presented for each indicator. Indicators are highlighted to reflect GCSI latent constructs (*GCSI dataset, 2019, N = 172*)

GCSI Latent Constructs	Indicator	Mean (Standard Deviation)	Median (Range)	***n***
Impact of the Crisis	Landmass affected - absolute	2.7 (1.3)	3 (1-5)	162
	Landmass affected - relative	3.6 (1.3)	4 (1-5)	162
	People living in the affected area - absolute	3.0 (1.3)	3 (1-5)	162
	People living in the affected area - relative	3.3 (1.5)	3 (1-5)	158
	People affected - absolute	2.4 (1.3)	2 (1-5)	153
	People affected - relative	2.5 (1.3)	2 (1-5)	149
	People displaced - absolute	2.6 (1.4)	3 (0-5)	133
	People displaced - relative	2.3 (1.4)	2 (0-5)	130
Complexity of the Crisis	Corruption perception	3.6 (0.5)	3.6 (1.7-4.5)	171
	Rule of law (WGI)	3.3 (0.6)	3.3 (1.6-4.8)	172
	Rule of law (BTI)	3.0 (0.8)	3.0 (0.3-4.5)	161
	Freedom in the world	3.0 (1.1)	3.1 (0.3-5.0)	172
	Total killed in all crisis	2.3 (1.5)	3 (0-5)	140
	Conflict intensity	3.5 (1.4)	3 (0-5)	172
	Gender inequality	3.3 (0.9)	3.5 (0.5-5.0)	151
	Income gini coefficient	1.9 (0.9)	2.1 (0.0-4.5)	149
	Ethnic fractionalisation	3.0 (1.4)	3.1 (0.0-5.0)	172
	Size of excluded ethnic groups	1.4 (1.6)	1 (0-5)	172
	Empowerment	2.8 (1.2)	2.6 (0.7-5.0)	168
	BTI - Democracy status	2.7 (0.8)	2.7 (0.5-4.3)	161
	Crisis affected groups	3.1 (1.4)	3 (1-5)	165
	Impediments to entry into country (bureaucratic and administrative)	0.7 (0.8)	0 (0-3)	161
	Restriction of movement (impediments to freedom of movement and/or administrative restrictions)	1.0 (1.1)	1 (0-3)	162
	Interference into implementation of humanitarian activities	0.9 (1.0)	1 (0-3)	162
	Violence against personnel, facilities and assets	0.5 (1.0)	0 (0-3)	164
	Denial of existence of humanitarian needs or entitlements to assistance	0.6 (0.9)	0 (0-3)	159
	Restriction and obstruction of access to services and assistance	1.3 (1.1)	1 (0-3)	161
	Ongoing insecurity/hostilities affecting humanitarian assistance	1.1 (1.2)	1 (0-3)	161
	Presence of mines and improvised explosive devices	1.1 (1.0)	1 (0-3)	156
	Physical constraints in the environment (obstacles related to terrain, climate, lack of infrastructure, etc.)	1.6 (1.1)	2 (0-3)	160
Conditions of the People	Total People in Need	2.4 (1.4)	2 (0-5)	137
	Current humanitarian conditions of total population in the affected area	3.0 (0.9)	3 (1-5)	137

We applied factor analysis to test whether the indicators in the GCSI dataset could be used to generate an estimate of crisis severity. Our approach had two primary steps: exploratory factor analysis (EFA), followed by confirmatory factor analysis (CFA). We removed all “pillar” aggregated values and only used the input data to generate our models. In the EFA model, we imputed median values of a given indicator if the observation was missing, while in the CFA model, we used maximum likelihood to address missing information. To assess whether these approaches influenced the final model estimates, we also ran the EFA model using case deletion for missing observations and the CFA model using multiple imputation (Additional file 1: Appendix 9). We found negligible differences between the results from these different approaches and those presented here (Additional file 1: Appendix 9).

#### EFA

We examined 32 of the 35 GCSI variables to assess their grouped correlation patterns. The EFA models suggested a 3- or 4-factor solution (Table 3). While the 5- and 6-factor solutions had greater cumulative variance explained. The proportion variance explained for each factor did not add substantive information to the model. This was also evident in the indicator factor loadings for these models, which showed more cross-loadings between indicators on factors with less than 10% proportion variance explained (see Additional file 1: Appendix 2 for factor loadings for 5- and 6-factor solutions).

**Table 3:** Exploratory Factor Analysis summary information for each factor solution identified: sums of squared loadings, proportion variance explained and cumulative variance (*GCSI dataset, 2019, N = 172*)

Factors	Three-Factor Solution			Four-Factor Solution			Five-Factor Solution			Six-Factor Solution		
	*Sums of Squared Loadings*	*Proportion Variance Explained*	*Cumulative Variance Explained*	*Sums of Squared Loadings*	*Proportion Variance Explained*	*Cumulative Variance Explained*	*Sums of Squared Loadings*	*Proportion Variance Explained*	*Cumulative Variance Explained*	*Sums of Squared Loadings*	*Proportion Variance Explained*	*Cumulative Variance Explained*
Factor 1	4.76	0.15	0.15	4.55	0.14	0.14	2.49	0.08	0.08	4.6	0.14	0.14
Factor 2	3.67	0.11	0.26	3.81	0.12	0.26	4.66	0.15	0.23	2.45	0.08	0.22
Factor 3	6.56	0.20	0.46	6.58	0.21	0.47	3.37	0.11	0.34	5.57	0.18	0.4
Factor 4	–	–	–	1.97	0.06	0.53	5.96	0.19	0.53	1.84	0.06	0.46
Factor 5	–	–	–	–	–	–	1.97	0.06	0.59	3.18	0.10	0.56
Factor 6	–	–	–	–	–	–	–	–	–	2.02	0.06	0.62

Additional examination of the factor loadings in 3- and 4- factor models highlighted three primary findings (Table 4). First, several indicators had factor loadings less than 0.30, which implies that they do not contribute to any of the factors. Second, indicator cross-loadings onto multiple factors were common, and thus, these indicators did not provide unique information. Finally, the indicators grouped into a pattern similar to sections of the GCSI conceptual framework. In both solutions, factor 1 was comprised of indicators related to societal constructs (and originally conceptualized as part of the ‘complexity of the crisis’), while indicators within the ‘impact of the crisis’ construct grouped together in factor 2. Factor 3 was comprised of indicators related to humanitarian access and safety; while the fourth factor was a further disaggregation of factor 2. Of note, the EFA results did not show that indicators related to ‘conditions of the people’ had mathematical importance. Indicators excluded from subsequent CFA models are shown in Table 4.

**Table 4:** Global Crisis Severity Index (GCSI) Indicators factor loadings for each Exploratory Factor Analysis (EFA) factor solution. Factor loadings are only presented if greater than the absolute value of 0.3 (*GCSI dataset, 2019, N = 172*)

Indicators	Three-Factor Solution			Four-Factor Solution			
	*Factor 1*	*Factor 2*	*Factor 3*	*Factor 1*	*Factor 2*	*Factor 3*	*Factor 4*
^a^Landmass affected - absolute		0.43	0.32		0.45	0.32	
Landmass affected - relative		0.86			0.86		
^a^People living in the affected area - absolute		0.49	0.37		0.49	0.33	
People living in the affected area - relative		0.93			0.93		
^a^People affected - absolute		0.51	0.38		0.53	0.35	
People affected - relative		0.74			0.75		
^b^People displaced - absolute			0.49			0.44	−0.41
^b^People displaced - relative		0.36			0.37		−0.33
Corruption perception	0.64			0.51			
^a^Rule of law (WGI)	0.56		0.35	0.42		0.42	0.47
Rule of law (BTI)	0.96			0.96			0.49
Freedom in the world	0.89			0.87			
Total killed in all crisis			0.72			0.73	
Conflict intensity			0.70			0.71	
Gender inequality	0.38						0.51
Income gini coefficient			−0.35			−0.31	0.41
^a^Ethnic fractionalisation							
^a^Size of excluded ethnic groups				0.34			−0.31
Empowerment	0.47			0.51			
BTI - Democracy status	0.93			0.95			
Crisis affected groups			0.54			0.54	
^a^Impediments to entry into country (bureaucratic and administrative)	0.36		0.35	0.46		0.31	−0.34
Restriction of movement (impediments to freedom of movement and/or administrative restrictions)			0.87			0.88	
Interference into implementation of humanitarian activities			0.67			0.65	
Violence against personnel, facilities and assets			0.57			0.58	
Denial of existence of humanitarian needs or entitlements to assistance			0.31	0.35			−0.39
Restriction and obstruction of access to services and assistance			0.80			0.79	
Ongoing insecurity/hostilities affecting humanitarian assistance			0.76			0.79	
Presence of mines and improvised explosive devices			0.62			0.64	
Physical constraints in the environment (obstacles related to terrain, climate, lack of infrastructure, etc.)	0.38						0.34
^a^Total People in Need		0.51	0.40		0.53	0.36	
^a^Current humanitarian conditions of total population in the affected area							

^a^Indicator excluded from subsequent CFA models

^b^Indicator excluded from CFA model with 4 latent constructs, but included in CFA with 3 latent constructs

#### CFA

We initially built four different CFA models to reflect the relationships identified with the factor loadings in the EFAs; each of the four models had an increasing number of latent constructs (from three constructs to six constructs).

**Fig. 2:**
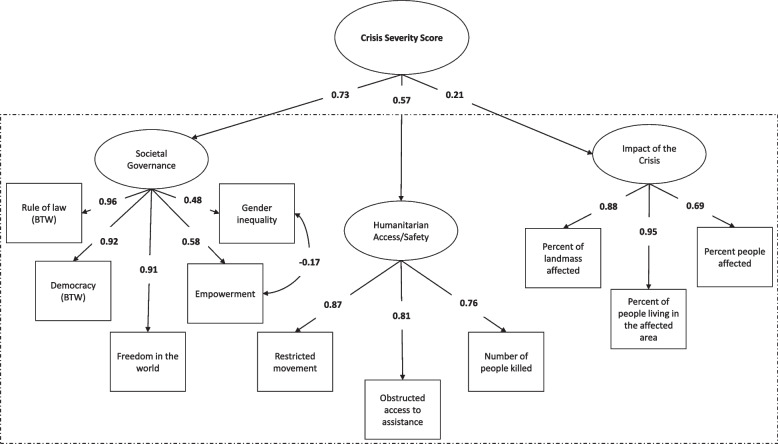
Second-order Confirmatory Factor Analysis (CFA), with factor loadings. The ovals reflect latent variables and the boxes reflect indicators. The dashed box contains the final first-order CFA

The CFA with three latent constructs (base model) was appropriately specified but showed poor fit (Table 5). Indicators were removed and covariances added to reflect the residual correlations of indicators across the dataset (see Additional file 1: Appendix 3 for correlation matrix) until the best model fit was generated (final model in Table 5). The final CFA contained 11 indicators: rule of law, democracy, freedom, gender inequality, empowerment, number of people killed, restricted movement, obstructed access to assistance, percent of landmass affected, people living in the affected area, people affected. We used this model to create a second-order CFA (Fig. 2). The model fit statistics of the second-order CFA were the same as the fit statistics of the final first-order CFA model (Table 5). In the second-order CFA, the magnitude of standardized factor-loading on the ‘societal governance’ latent construct had the strongest association with the latent construct of ‘crisis severity’ (0.73), followed by the ‘humanitarian access/safety’ construct (0.56).

**Table 5:** Fit statistics for first-order Confirmatory Factor Analysis (CFA) models: Chi-squared goodness of fit test statistic, Comparative Fit Index (CFI), Tucker-Lewis Index (TLI), and Root Mean Square Error of Approximation (RMSEA) Index (*GCSI dataset, 2019, N = 172*)

Model fit statistics	Base model	Final Model
Chi-squared goodness of fit (degrees of freedom)	735 (206)	107 (40)
CFI	0.77	0.94
TLI	0.75	0.92
RMSEA	0.12	0.10

The CFA with four latent constructs had a non-positive covariance matrix when a second order latent variable was added. No solutions were found for the 5 or 6 latent variable models.

#### Severity score

**Fig. 3:**
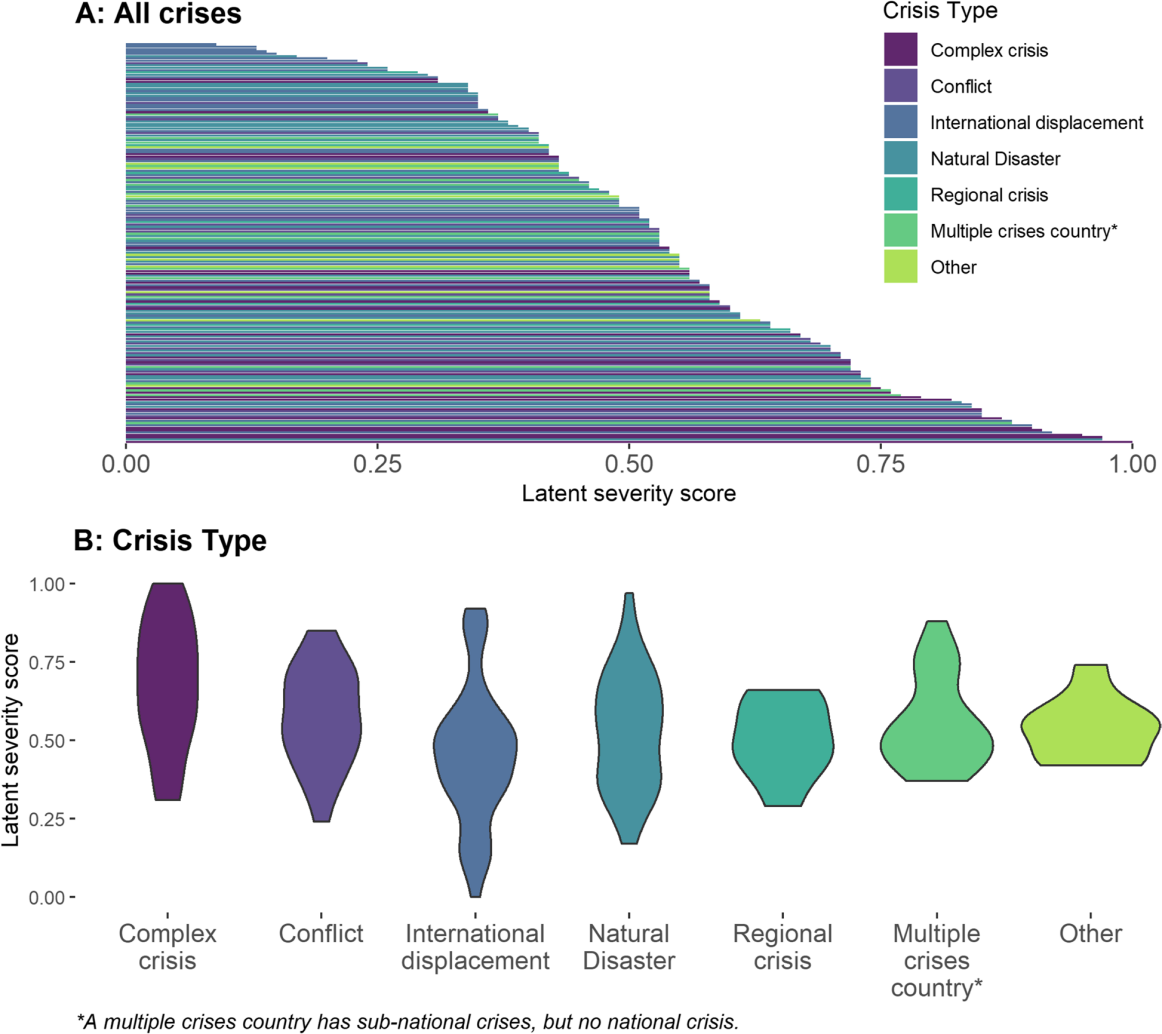
The distribution of latent crisis severity scores. Figure 2A shows all crises (*n* = 172) and Fig. 2B is stratified by crisis type

We used the final CFA model to generate normalized severity scores for each crisis (which range between 0 and 1 to represent low to high severity). The mean and median latent severity score for all crises were similar, at 0.53 and 0.54, respectively. Severity scores were highest in complex crises and fell within the upper two-thirds of all scores (Fig. 3). Regional crises, conversely, had a lower mean severity score. These types of crises fell into the bottom two-thirds of the range. Crises in countries that had a mean severity score of greater than 0.90 included Syria, Somalia, Yemen, and The Democratic People’s Republic of Korea, whereas countries with mean severity scores less than 0.10 included Costa Rica and Brazil.

## Discussion

Our analyses show that crisis severity can be measured best through use of only 11 of the 35 GCSI indicators. Put another way, 24 of the 35 indicators used in the GCSI model do not contribute useful numerical information. In our final model, the strongest predictors of severity were a suite of indicators related to social structure/governance of a given nation state (rule of law, freedom, gender inequality, and empowerment), followed by indicators that were proxy measurements of humanitarian access/safety (number of people killed, restricted movement, and obstructed access to assistance). Weaker, although still relevant, predictors were related to the crisis impact on people and the environment. Overall, this analysis suggests that most of the key variables to estimate the severity of humanitarian need can be assessed globally, can be collected in a comparable way from one country to another, and do not depend on sudden changes to local conditions that would be unavailable to those making the calculations. In short, despite changing conditions and limited or imperfect information, we can use existing data sources to make reasonable estimates of severity around the world. Refinements to the existing GCSI model will make it easier and more reliable to make these estimates.

Holistically, the 11 selected indicators suggest that fragile states with limited accessibility for humanitarian actors have worse humanitarian conditions. This final model aligns with humanitarian actors’ experiences. Indeed, good governance is intrinsically related to avoiding or mitigating a humanitarian crisis [12]. We tested the role of governance in our models (see Additional file 1: Appendix 8) and found it to be a key latent constructure of severity, but only when crisis related variables were also included in the model. Broadly, economic and political stability are key components to this success, with inequality between social groups cited as a driver of crises and conflicts [25]. It is unsurprising that humanitarian practitioners call for more robust inclusion of conflict early warning into preparedness systems for humanitarian crises [26]. Indeed, considerable funding has been provided to post-conflict states for democracy development and peacebuilding, albeit with mixed success [27, 28]. Ample evidence supports these patterns, as data from the last 15 years show most humanitarian crises are re-occurring in the same countries, many of which are fragile states [13]. Chad, the Central African Republic, the Democratic Republic of Congo, Somalia, and Sudan have all had 15 crises between 2005 and 2015.

Beyond governance, access to reach those in need is also important to reducing crisis severity. Humanitarian access, the ability to reach the most vulnerable, can be limited through various mechanisms. Restricted movements, which are common in conflicts and complex humanitarian crises, inhibit connections between aid workers and communities [29]. Access can also be reduced through violence and obstruction. Within armed conflicts, bureaucratic and security constraints, and violence against aid workers and facilities distributing aid, have been cited as rationale for greatly reduced humanitarian access [29, 30]. For example, in the Syrian crisis, which is considered one of the worst in the world by humanitarian experts, UNOCHA reported that 1.1 million people were in need of humanitarian assistance in hard-to-reach-places in 2018; during this same year, access was inhibited by 142 attacks on health facilities, with 102 people dead and 189 injured [31]. Thus, it is not surprising that our model results give weight to indicators reflecting the quality of humanitarian access (e.g., restricted movements, obstructed access, and number of people killed) for a given crisis.

Importantly, our final model differs from the original GCSI in two fundamental ways. First, we presented a parsimonious model, which removed 24 GCSI indicators. Using the reduced set of 11 indicators, the model showed acceptable fit, but had slightly higher error than the standard cut points; however, some debate exists on the usefulness of applying a single heuristic to assess model fit within factor analysis [32]. The original GCSI was calculated using inconsistent approaches, and notably, does not account for basic statistical properties of correlated data. The high correlation in the dataset inhibits meaningful interpretation of combined values from the indicators. Second, we removed an entire GCSI pillar (‘conditions of the people’) as a result of insights from the EFA models, which has programmatic significance. Indeed, the data underlying the excluded indicators are routinely collected to estimate the number of people in need of humanitarian assistance. Given the strong value of these indicators to practitioners, we re-ran the final model and included these two indicators as standalone independent variables (Additional file 1: Appendix 5). Of note, we did not include the two indicators as latent constructs, as our EFA analyses showed that they were not correlated. This sensitivity analysis suggested that a model including the *number of people affected* indicator has comparable model fit and yields similar severity scores to the second-order CFA model.

Our analysis, however, is limited by the data available for inclusion. First, the index includes a combination of static and dynamic variables. It is possible that static variables, such as those used to estimate social structure/governance are distal determinants of a crisis, rather than proximal measures. Additionally, we used population average data, which masks any disparities experienced within a population. Several population groups, namely, children, women, and the disabled, have worse crisis-related health outcomes than the rest of the population. Moreover, data from humanitarian crises are difficult to obtain, highly inaccurate, and highly correlated. While our sensitivity analyses assessing data quality suggest that our final model contained data that was no more or less reliable than the indicators excluded (Additional file 1: Appendix 6), we cannot account for the lack of precision within the dataset. We included two indicators based on expert assessment of qualitative information (restricted movement, and obstructed access to assistance), which may be subject to imprecision or bias. Likewise, mortality estimates, which we also included in the final model, have been contested for accuracy in past crises [33, 34]. Additionally, the indicator for ‘relative people living in the affected area’ is highly correlated with many of the other variables in the final model. In an ideal scenario, this indicator would be removed from the model, however, when it was, the models did not converge. Thus, one limitation of retaining the variable is a slightly higher error than desired. Finally, we are limited in our ability to test the generalizability of the model given the small sample size and lack of additional data for testing. Nevertheless, our comparison of the model fit statistics and factor loadings to suggest that the model performance is consistent and unlikely overfit to the data (Additional file 1: Appendix 10).

Importantly, a gold standard for crisis severity is unavailable to validate our model results and out-of-sample data were not available to assess predictions. In lieu of traditional validation, we compared the latent severity scores to the original GCSI scores (Additional file 1: Appendix 7). This robustness check suggested that the latent severity score may be a closer measure to true crisis severity than the original GCSI. Despite the limitations with data availability and independent data source for validation, we emphasize that this work is a first step towards improving crisis severity measurement. Because the calculations are derived from a model that weights indicators based on their correlations, estimating severity for a new crisis would require re-running the final CFA. Further research is needed to assess the feasibility of linking this framework with a field friendly application for humanitarian actors after additional analyses have been conducted.

## Conclusion

UN-coordinated humanitarian responses are lasting longer [35], with the average 2005 response ongoing for about 4 years compared to the 2017 response of 7 years. Meanwhile, human and financial resources for humanitarian response are limited. More complicated responses, coupled with calls for increased resources, emphasize the need for objective tools to guide resource allocation. Indeed, a metric of crisis severity can add powerful contributions to determine priorities for humanitarian response, highlighting whether severity and subsequent aid/response align. However, a metric of crisis is only useful if the metric is scientifically robust. Our work is a first step in refining an existing framework to quantify crisis severity. We suggest three additional areas of needed exploration. As presented here, we recommend all future iterations of modeling crisis severity consider severity as a multi-faceted construct. In doing so, practitioners should strive to create a parsimonious model. Inherently, humanitarian data are subject to high levels of uncertainty, and nonparsimonious models may further limit clear interpretation of severity within this context. Additionally, we recommend that future work consider longitudinal metrics of severity, as crises change within a given location over time. After further testing this model with additional crises, opportunities for converting model output to a dashboard or application for humanitarian actors should be explored. At the time of writing this manuscript, the current GCSI estimates were available in a large spreadsheet available at https://data.humdata.org/. They are now also available on the ACAPs website (https://www.acaps.org/methodology/severity) in an interactive dashboard and available to be accessed through an Application Programming Interface (API; https://api.acaps.org/). This interface provides a blueprint for merging robust statistical output with information needed by data users. With these recommendations in place, humanitarian actors can apply the humanitarian principle of impartiality when determining where need is the greatest and to best respond to crises.

## Supplementary Information

**Additional file 1: Appendix 1.** The Global Crisis Severity Index (GCSI) Conceptual Framework and Estimation. **Appendix 2.** Factor loadings for 5-factor and 6-factor Exploratory Factor Analysis (EFA) solutions. **Appendix 3.** Correlation Matrix for First-Order Confirmatory Factor Analysis (CFA). **Appendix 4.** R code for primary analyses. **Appendix 5.** Inclusion of “Conditions of People Affected by the Crisis”. **Appendix 6.** Indicator reliability. **Appendix 7.** Sensitivity analyses for derived latent severity scores. **Appendix 8.** The role of state fragility in the final model. **Appendix 9.** Missing data exploration. **Appendix 10.** Further assessment of model fit.

## Data Availability

The datasets generated and/or analysed during the current study are available in the *GCSI Database Beta Version – November 2019* repository, https://data.humdata.org/dataset/inform-global-crisis-severity-index

## Abbreviations

UNOCHA: United Nations Office for the Coordination of Humanitarian Affairs
UN: United Nations
GCSI: Global Crisis Severity Index
EFA: Exploratory Factor Analysis
CFA: Confirmatory Factor Analysis
CFI: Comparative Fit Index
TLI: Tucker-Lewis Index
RMSEA: Root Mean Square Error of Approximation
API: Application Programming Interface
